# Retention of an Intrauterine Pregnancy After the Removal of a Heterotopic Cervical Twin Pregnancy

**DOI:** 10.7759/cureus.94990

**Published:** 2025-10-20

**Authors:** Nikolaos Antonakopoulos, Panagiota Tzela, Leonidas Antonakis, Georgios Adonakis

**Affiliations:** 1 Department of Obstetrics and Gynecology, School of Health Sciences, University of Patras, Patras, GRC; 2 Department of Midwifery, School of Health and Care Sciences, University of West Attica, Athens, GRC

**Keywords:** case report, cervical pregnancy, ectopic pregnancy, heterotopic pregnancy, ivf

## Abstract

Cervical pregnancy is an uncommon form of ectopic pregnancy, and when combined with a coexisting intrauterine gestation, it represents a particularly rare and challenging clinical scenario. We report the case of a 46-year-old woman with a history of multiple IVF attempts who conceived after the transfer of two blastocysts. At eight weeks, ultrasound revealed a heterotopic triplet pregnancy: a monochorionic twin cervical ectopic pregnancy alongside a singleton intrauterine gestation. Cardiac activity was present in all fetuses. After counseling, selective evacuation of the cervical pregnancy was performed via dilatation and curettage, followed by prophylactic cervical cerclage to prevent iatrogenic insufficiency. The intrauterine pregnancy progressed uneventfully until 32 weeks, when placental insufficiency developed. Delivery by cesarean section at 34 weeks resulted in a healthy male neonate weighing 2100 g, discharged after a brief NICU stay. This report highlights an exceptionally rare case of heterotopic cervical twin pregnancy coexisting with a viable intrauterine pregnancy. It emphasizes the importance of early diagnosis, individualized management, and multidisciplinary care to optimize both maternal safety and fetal outcome.

## Introduction

Cervical pregnancy has a reported incidence of 1:1000 to 1:18,000 pregnancies, accounting for less than 1% of ectopic pregnancies, and it is characterized by implantation in the uterine endocervical canal [1]. Heterotopic pregnancy, the coincidence of an orthotopic intrauterine pregnancy and an ectopic pregnancy, is extremely uncommon, with an estimated incidence of one in 30,000 natural conceptions. Heterotopic pregnancy with a cervical ectopic component is even more rare. Contributing factors include previous cesarean section, dilation and curettage, and assisted reproductive technologies (ARTs) [2], with the latter raising the incidence significantly to 0.15% and even above [3]. To our knowledge, the case of a heterotopic cervical twin pregnancy with retention of the orthotopic intrauterine pregnancy has never been reported in the past. This exceptionally rare case presents unique clinical challenges, given that the cervical location of the ectopic pregnancy is particularly concerning due to its higher risk of maternal complications, such as severe vaginal bleeding and preterm labor [4]. The significantly increased risk of bleeding might eventually result in hysterectomy or even death of patients; therefore, early diagnosis and treatment are essential, especially for those women who wish to preserve their fertility [5].

Given the diagnostic complexities associated with cervical heterotopic pregnancies, early identification through high-resolution transvaginal ultrasound and Doppler imaging is crucial to prevent complications. Despite improvements in ultrasound technology, early diagnosis remains challenging due to the asymptomatic nature of this ectopic pregnancy in the initial stage. Moreover, no globally established guideline exists for its management. Several case reports in the literature describe different methods for the treatment of heterotopic cervical pregnancy, including aspiration, cervical curettage, Foley catheter placement, and local injection of potassium chloride or methotrexate. Once diagnosed, management strategies must be carefully selected to balance maternal safety and fetal viability.

Here, we present the exceptionally rare case of a heterotopic triplet pregnancy, involving a monochorionic twin cervical ectopic pregnancy and a singleton intrauterine pregnancy, in which, after successful removal of the cervical pregnancy, we managed to preserve the orthotopic intrauterine pregnancy, leading to a healthy live-born late preterm neonate. We also present a literature review on the topic concerning usual management. By documenting the diagnostic process, management challenges, and treatment outcomes, this report contributes to the growing body of literature on cervical heterotopic pregnancies and underscores the importance of individualized care approaches.

## Case presentation

**Figure 1:**
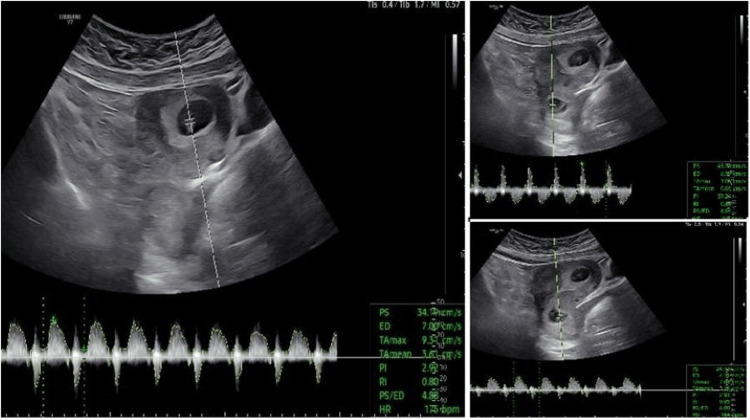
Heterotopic triplet pregnancy, involving a monochorionic twin cervical ectopic pregnancy and a singleton intrauterine pregnancy A heart rate was documented in all three fetuses.

This was a 46-year-old woman with multiple IVF attempts, who finally achieved pregnancy after IVF with two-blastocyst transfer. Unfortunately, already at the eighth week, it was found that both blastocysts were implanted, but one of them was located in the cervical canal after splitting in half, creating a heterotopic twin cervical pregnancy. A heart rate was documented in all three fetuses (Figure 1). There were no symptoms or clinical signs of pregnancy complications until that point. Despite the unfavorable prognosis and the indication for termination of the whole pregnancy, after detailed consultation, the couple opted for removal of the cervical pregnancy and maintenance of the orthotopic pregnancy.

**Figure 2:**
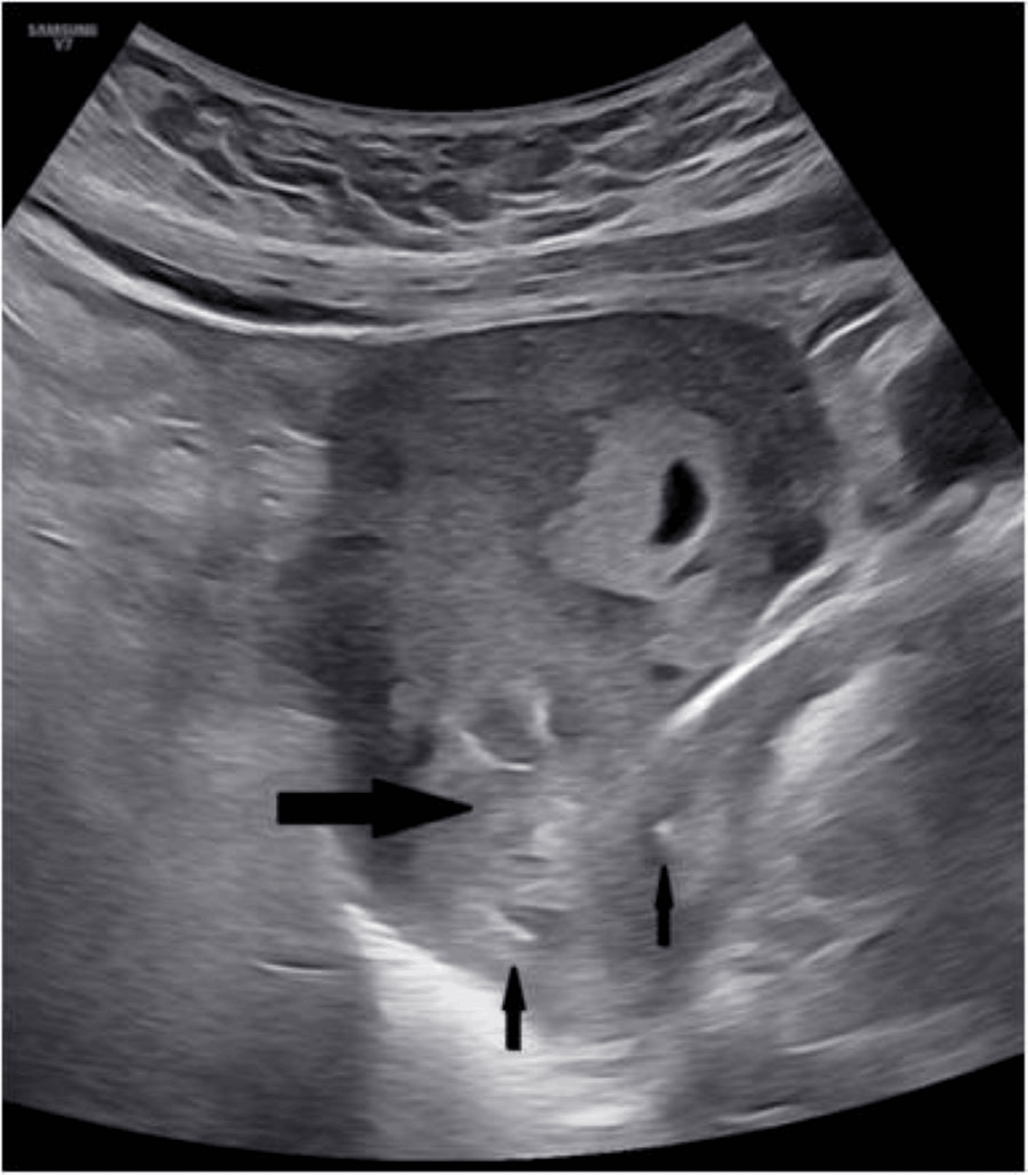
Sonographic appearance of the uterus after curettage The thick black arrow represents the upper cervical canal where the twin ectopic pregnancy was implanted, whereas the thin black arrows indicate the cervical cerclage suture.

**Figure 3:**
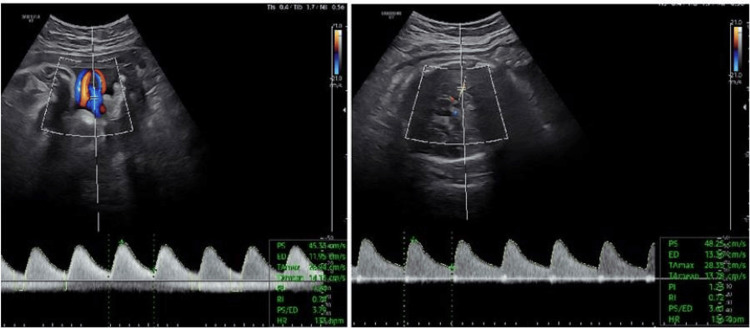
Umbilical and middle cerebral artery Doppler indicating fetal blood redistribution (CPR = 1) CPR, cerebroplacental ratio

A successful cervical dilatation and evacuation was performed, followed by a cervical cerclage, due to the expected post-surgical cervical insufficiency (Figure 2). The procedure was done under short general anesthesia and trans-abdominal sonographic guidance, with estimated minimal blood loss. Progesterone support was already given vaginally by the IVF specialist and was continued until 14 weeks of gestation. The woman had a regular monthly follow-up, and the breech singleton pregnancy continued uneventfully until the 32nd week, when placental insufficiency occurred and a cesarean section was finally performed at 34 weeks, due to fetal circulation redistribution (Doppler cerebroplacental ratio less than 1), after the administration of steroids (Figure 3). A healthy male neonate weighing 2100 g was born. After a short stay in the NICU, he was discharged home.

Written informed consent for the publication of this case report was obtained from the patient.

## Discussion

Our case illustrates the unique challenges of managing a heterotopic cervical twin pregnancy coexisting with a viable intrauterine gestation. The patient’s history of multiple IVF attempts aligns with existing reports that link ARTs to an increased risk of heterotopic pregnancies. Fan et al.’s review found that more than 80% of reported cases occurred after ART, underscoring this association [4]. Embryo reflux, cervical manipulation during embryo transfer, the altered hormonal environment during ovarian stimulation, and the detrimental effect of supra‐physiological hormone levels on endometrial receptivity may be hypothesized as the underlying mechanisms [6,7]. The rarity of a monochorionic twin cervical pregnancy makes this case exceptional, as most published cases describe a singleton cervical gestation.

The clinical presentation of our patient, with no symptoms or clinical signs and ultrasound diagnosis at eight weeks with present cardiac activity in all fetuses, is consistent with previous findings that transvaginal ultrasound remains the gold standard for early detection and highlights the value of the early pregnancy scan in all IVF pregnancies as a routine. MRI was not required, as the diagnosis was clear and without complicating features. In terms of management, medical therapy with methotrexate or potassium chloride was contraindicated due to the coexisting intrauterine pregnancy. Thus, selective evacuation of the cervical pregnancy by curettage was chosen, followed by prophylactic cervical cerclage. This combination was tailored to minimize the risk of cervical insufficiency and to preserve the intrauterine pregnancy. The outcome, delivery at 34 weeks after the onset of placental insufficiency, demonstrates that favorable neonatal survival is possible despite the complexity of such cases.

According to the literature, most patients with an ectopic cervical pregnancy present with painless vaginal bleeding during the first trimester, with or without associated pelvic pain [8]. Doppler ultrasound and magnetic resonance imaging are commonly used diagnostic methods. Transvaginal ultrasound remains the gold standard for diagnosis, and hopefully, 70% of the cases are diagnosed early between five and eight weeks of gestation, highlighting the critical window for early detection [4]. The sonographic diagnostic criteria include an echo‐free uterine cavity, hour‐glass uterine shape, ballooned cervical canal, gestational sac in the endocervix, located below the internal os, and closed internal orifice [9]. Cervical pregnancy must always be distinguished from a miscarriage in progress, an intrauterine pregnancy that slips to the cervix. The latter can be visualized with the “sliding sign” by transvaginal ultrasound, where the gestational sac slides to the top of the cervical canal when gently pressing the vaginal ultrasound probe [10]. MRI is rarely used as a supplementary examination for complex infiltrating cases or when ultrasound resolution is hampered [11].

Management of heterotopic cervical pregnancies remains highly individualized, as no standardized protocol exists. Published options include systemic or local methotrexate, potassium chloride injection, aspiration, dilatation and curettage, hysteroscopy, uterine artery embolization, cervical cerclage, and, more recently, microwave ablation. The choice depends on gestational age, viability of the cervical pregnancy, presence of intrauterine pregnancy, maternal hemodynamic status, and the couple’s fertility goals. As serum β‐HCG levels rise, the risk of medical treatment failure and/or massive hemorrhage also increases [12].

Fan et al.’s review of 21 cases highlighted the heterogeneity of practice [4]: 62% were treated surgically (aspiration, hysteroscopy, embolization, forceps extraction, and cerclage), 14% medically (methotrexate, potassium chloride, and hyperosmolar solutions), and 24% with combined approaches. The continuation of intrauterine pregnancy was achieved in over 70% of cases, and maternal hysterectomy was rarely required. Delivery was most often by cesarean section, though not always for indications related to the cervical pregnancy. More recently, Schivardi et al. reported the use of microwave ablation as a novel fertility-preserving option, broadening the therapeutic spectrum [13].

This is not the first case of successful treatment of an extrauterine pregnancy with the prosecution of the intrauterine pregnancy up to the third trimester [14]; however, the cervical ectopic twin component makes our case unique and extremely challenging. Our management contributes to this growing body of evidence by demonstrating that surgical evacuation with adjunctive cervical cerclage can successfully preserve intrauterine pregnancy even in the context of a cervical twin gestation. While encouraging, individualization remains critical, as clinical diversity and the potential for massive hemorrhage prevent a one-size-fits-all approach. Counseling, multidisciplinary input, and shared decision-making with the couple are essential to optimizing both maternal safety and fetal viability.

## Conclusions

Heterotopic cervical pregnancy is a rare but high-risk obstetric condition that demands early recognition and carefully tailored management. The increasing incidence, particularly among women undergoing ARTs, underscores the need for heightened clinical awareness. This case highlights the further rarity and complexity of managing a heterotopic cervical twin pregnancy coexisting with an orthotopic intrauterine pregnancy. The successful outcome following selective surgical evacuation of the cervical pregnancy and the subsequent preservation of the intrauterine fetus underscores the importance of individualized care, early diagnosis, and multidisciplinary management. While there are no established guidelines for such cases, this report provides valuable insight into a potential treatment strategy that balances maternal safety and fetal viability. Given the high risk of complications, such decisions must always be approached with thorough counseling and informed consent. As clinical experience and novel techniques expand, further accumulation of similar cases is essential to guide future recommendations and improve outcomes in this exceptionally rare clinical scenario.
